# Microbiological quality of drinking water from dispensers in Italy

**DOI:** 10.1186/1471-2180-10-19

**Published:** 2010-01-26

**Authors:** Giorgio Liguori, Ivan Cavallotti, Antonio Arnese, Ciro Amiranda, Daniela Anastasi, Italo F Angelillo

**Affiliations:** 1 Department of Public, Clinical and Preventive Medicine, Second University of Naples, Naples, Italy

## Abstract

**Background:**

Water coolers are popular in office buildings and commercial stores and the quality of this source of drinking water has the potential to cause waterborne outbreaks, especially in sensitive and immunocompromised subjects. The aim of this study was to determine the quality of water plumbed in coolers from commercial stores in comparison with tap water in Italy.

**Methods:**

For each sample, microbial parameters and chemical indicators of contamination were evaluated and information about the date of installation, time since last ordinary and extraordinary maintenance of water coolers was collected.

**Results:**

In all samples the chemical parameters (nitrite, ammonium, free active chlorine residual) did not exceed the reference values of the drinking water regulation; the pH value in 86.8% samples of the carbonated waters was lower than the reference limit. The microbiological results indicated that the bacteria count at 22°C and 37°C was higher than the required values in 71% and 81% for the non-carbonated water and in 86% and 88% for the carbonated one, respectively. *Enterococcus *spp. and *Escherichia coli *were not detected in any of the water samples. *Pseudomonas aeruginosa *was found in only one sample of the tap water and in 28.9% and 23.7% of the non-carbonated and carbonated water samples, respectively. No statistically significant differences in bacterial counts at 22°C and 37°C have been found between the non-carbonated and carbonated water from the sampled coolers in relation with the time since the last filter was substituted. The bacteriological quality of tap water was superior to that of non-carbonated and carbonated water from coolers.

**Conclusion:**

The results emphasize the importance of adopting appropriate routinely monitoring system in order to prevent or to diminish the chances of contamination of this water source.

## Background

It is well known that the quality and safety of the drinking water continues to be an important public health issue [1,2], because its contamination has been frequently described as responsible for the transmission of infectious diseases that have caused serious illnesses and associated mortality worldwide [3-6]. Clearly, point-of-use water quality is a critical public health indicator [2].

Over the past decade, there has been a markedly increase in the consumption of water derived from different sources in place of tap water for drinking use in many regions of the world. One of these alternative sources is the water from dispensers, which is popular mainly in office buildings and commercial stores, that are often presented as systems that are able to improve some characteristics of water and easy to use and to maintain. However, concerns have been sometimes raised about the quality of this source due to its potential to cause waterborne outbreaks associated with drinking water, particularly in sensitive and immunocompromised populations [2].

International drinking water-quality monitoring programs have been established in order to prevent or to reduce the risk of contracting water related infections. In Italy, the water for human consumption, including the water coming from dispensers, according to the European Community Directive guidelines, is required to be free from any pathogenic microorganism as well as chemical contaminations, which may be hazardous to the human health [7,8].

To the best of our knowledge, very few studies have been conducted to this end dealing with the quality of drinking water from coolers [9-12]. Therefore, the objective of the present study was to investigate the chemical and microbiological quality parameters of drinking water plumbed in water coolers in commercial stores in an area of Southern Italy.

## Methods

The data for this study were collected, during the period between February 2005 and September 2007, from the alphabetical list of the commercial stores located in the geographic area of the city of Naples, in the South of Italy. From this list, 41 stores were selected using a simple random sampling technique.

Before the study, as part of the process of informed consent, all selected commercial stores received an envelope with a letter informing that a research project was being conducted and describing the study, the voluntary nature of participation, and assurance of privacy and anonymity. For each participant, information about the date of installation, time since last ordinary and extraordinary maintenance of water coolers was collected with a self-administered questionnaire. The water coolers were produced by different companies, all were supplied from tap water and at the sampling time the mechanism of cooling was functioning. At the end of the study, each store received the results of the microbiological and chemical quality parameters investigated.

### Collection of water samples

Water samples were collected from coolers (carbonated and non-carbonated water) and tap water corresponding to the tap water used for the cooler from the same store. Each sample was analyzed for total viable count (TVC), qualitative microbial indicators, and chemical parameters of organic contamination. Two litres of each water sample were collected without flushing before sampling and sterilizing the outer surfaces of the faucets. Water samples were collected in sterile sample bottles containing sodium thiosulfate (100 mg/L). Samples were transported on blue ice in an insulated, double-walled container to the laboratory for analysis and primary isolation within 6 hours of sampling.

All analyses were performed according to the current Italian [7] and European regulations on drinking water for human consumption [8]. The reference values for the water in order to be declared potable are the following: *Enterococcus *spp., *Escherichia coli*, and *Pseudomonas aeruginosa *should not be detectable in 100 ml; TVC less than 100 and 20 colony forming units (CFU) per mm at 22°C and 37°C, respectively; nitrite and ammonium less than 0.5 mg/L; free chlorine comprised between the values of 0.2 and 0.8 mg/L; and pH between the values of 6.5 and 9.5.

### Microbiological parameters

The microbiological analyses of all water samples were conducted as follows:

1) TVC: 1 mL of each sample was included with 20 mL of Water Plate Count agar (bioMérieux Italia) and two sets of plates were prepared for all samples with each sample diluted until 10^-3 ^on three dishes. One set was incubated at 22°C for 72 hours and the other set at 37°C for 48 hours (prEN ISO 6222). All colonies were counted as CFU/mL of the water sample;

2) *Escherichia coli*: 250 mL of each sample was filtered through a 0,45 μm cellulose membrane filter, placed on Tergitol 7 TTC agar (bioMérieux Italia), and plates were incubated at 37°C for 18/24 hours (ISO 9308-1). The species identification was conducted using standardized identification system API 20E (bioMérieux Italia);

3) *Enterococcus *spp.: 250 mL of each sample was filtered through a 0,45 μm cellulose membrane filter, placed on Slanetz-Bartley agar (bioMérieux Italia), and plates were incubated at 37°C for 48 hours. If typical colonies (red/brown/pink) were present, the membrane was transferred on pre-warmed (44°C) plates of Bile Aesculina Azide agar (bioMérieux Italia) and incubated at 44°C for 2 hours (ISO 7899-2). Typical brown/black colonies were identified as *Enterococcus *spp. using standardized identification system API 20 Strep (bioMérieux Italia);

4) *Pseudomonas *spp.: 250 mL of each sample was filtered through a 0,45 μm cellulose membrane filter, placed on Pseudomonas CN agar (Cetrimide-Nalidixic Acid, bioMérieux Italia), and plates were incubated at 37°C for 48 hours, blue/green colonies were isolated on Plate Count agar (bioMérieux Italia) at 37°C for 24 hours, and after the oxydase test (bioMérieux Italia), the species identification was conducted using standardized identification system API 20NE (bioMérieux Italia) (prEN ISO 12780);

5) Other microorganisms: singles colonies growing on Tergitol 7 TTC agar (bioMérieux Italia) were transferred on McConkey agar (bioMérieux Italia), and plates were incubated at 37°C for 24-48 hours; after the oxydase test (bioMérieux Italia), the species identification was conducted using standardized identification systems API 20E/20NE (bioMérieux Italia).

### Chemical analyses

#### pH

The pH was determined electrometrically by using the technique recommended in the Standard Methods [13].

#### Residual free chlorine

The residual free chlorine content was measured using the *N*,*N*-diethyl-*p*-phenylenediamine (DPD) colorimetric method at the time of sample collection (colorimetric DPD method; Microquant; Merck, Darmstadt, Germany) [13].

#### Ammonium

For ammonium ions determination, 50 mL of the water sample and the calibration samples were mixed with 1 mL of a potassium tetraiodiomercurate solution. After 20 minutes reaction time at room temperature in NH_3_-free atmosphere, the solution was examined photometrically at a wavelength of 420 nm in cuvettes of appropriate path length (IRSA-CNR, Rome, Italy).

#### Nitrite

For nitrite ion determination, 50 mL of the water sample and the calibration samples were mixed with 2 mL of a freshly prepared mixture of equal parts of sulphanilic acid solution and 1-naphthylamine solution. After 2 hours at 20°C in darkness the extinction at 530 nm was measured [14].

### Statistical analysis

Basic descriptive summaries were used to describe measures of central tendencies and dispersion of water characteristics and microbial concentrations. The χ^2 ^and the Fisher's exact test were used to determine whether statistically significant differences existed in the contamination levels between coolers water (non-carbonated and carbonated) and tap water. The simple linear regression was used to determine whether statistically significant associations existed between the bacterial counts of the coolers water (non-carbonated and carbonated) and the time since the last filter was substituted. Statistical significance was assessed using two-sided tests with *p*-values of ≤ 0.05. Analyses were performed using the statistical package Stata [15].

## Results

Of the 41 randomly selected commercial stores, 38 agreed to participate for a response rate of 94.7%. The time since the last maintenance of water coolers, comprehensive of filter substitution, in the participating stores ranged between 1 and 24 months.

A description of the data regarding microbiological characteristics of drinking water dispensed by the sampled water from coolers and tap according to the Italian legislation is provided in Additional file 1. It should be noted that *Enterococcus *spp. and *Escherichia coli *were not detected in any of the water samples. In 17% of the samples of tap water after incubation at 22°C and 37°C the number of aerobic bacteria was higher than the stated drinking water limits for TVC of < 100 CFU/mL and < 20 CFU/mL, respectively. *Pseudomonas aeruginosa *was found in only one sample of the tap water and in 28.9% and 23.7% of the non-carbonated and carbonated water samples, respectively. The microbiological results for the water coolers indicated that the total bacteria counts at 22°C and 37°C was higher than the required values in 71% and 81% for the non-carbonated water and in 86% and 88% for the carbonated one, respectively. The overall mean bacteria counts at 22°C and 37°C in the water samples were respectively 102.9 CFU/mL and 86.3 CFU/mL for the tap, 569.7 CFU/mL and 331.8 CFU/mL for the non-carbonated, and 542.1 CFU/mL and 355.9 CFU/mL for the carbonated.

The results of the statistical analysis conducted to determine whether differences exist among the three different types of water with regard to microbial measures showed no significant difference between the number of microorganisms recovered from the non-carbonated and carbonated water from coolers for the bacteria count at 22°C (χ^2 ^= 2.55, *p *= 0.18) and at 37°C (χ^2 ^= 0.82, *p *= 0.55), and for *Pseudomonas aeruginosa *(χ^2 ^= 0.26, *p *= 0.8), respectively. The tap water was always of excellent bacteriological quality and it was superior than the water from coolers. Indeed, a statistically significant higher proportion of positive microbial counts has been recorded for both bacterial counts at 22°C and 37°C in the non-carbonated (χ^2 ^= 25.55, *p *< 0.0001; χ^2 ^= 34.73, *p *< 0.0001) and carbonated (χ^2 ^= 40.07, *p *< 0.0001; χ^2 ^= 42.95, *p *< 0.0001) waters compared with the tap water. The number of positive samples for *Pseudomonas aeruginosa *was significantly higher in the non-carbonated (Fisher's exact test *p *= 0.003) and carbonated (Fisher's exact test *p *= 0.015) water coolers samples compared with the samples of tap water. Moreover, the results of the simple linear regression analysis showed that no statistically significant associations between bacterial counts at 22°C and 37°C either for the non-carbonated (*p *= 0.16 and *p *= 0.15) or the carbonated water (*p *= 0.21 and *p *= 0.14) from the sampled coolers in relation with the time since the last filter was substituted.

Other microorganisms were isolated from 6 (20%), 25 (65.8%), and 27 (71.1%) samples of the tap, non-carbonated, and carbonated waters. The bacteria were identified mainly to be *Pseudomonas *species, which was recovered respectively from 6 (20%) samples of the tap water and from 19 (50%) samples either of the non-carbonated or the carbonated waters, and the mean concentrations were 48.3 CFU/mL, 241.5 CFU/mL, and 137.2 CFU/mL, respectively. Species of *Stenotrophomonas*, *Pasteurella*, *Enterobacteria*, and *Flavobacterium *were also isolated mainly from the non-carbonated or the carbonated waters.

With regard to the chemical parameters, in all samples the nitrite, ammonium, and free active chlorine residual did not exceed the reference values of the drinking water regulation. The mean average values of the three parameters for the tap water were 0.06 mg/L (range 0.001-0.15) for nitrite and 0.08 mg/L (range 0.01-0.25) for both ammonium and free active chlorine residual; whereas, for the carbonated and non-carbonated waters the average values were 0.076 (range 0-0.025) and 0.06 mg/L (range 0-0.025) for nitrite, 0.08 (range 0-0.3) in both waters for ammonium, and 0.3 (range 0.2-0.4) and 0.29 mg/L (range 0.2-0.4) for free active chlorine residual, respectively. Finally, the pH of the tap and non-carbonated waters did not exceed the reference value and both means were 7.8 ranging from 6.8 and 8.4, whereas for the carbonated the vast majority of the samples (86.8%) had a value lower than the reference limit with an overall mean of 6 and a range of 5.2 and 6.8.

## Discussion

This study sought to determine the quality of drinking water dispensed by water coolers from commercial stores in comparison with tap water in the geographic area of Naples, Italy.

In this investigation, the microbiological quality of the drinking water was satisfactory for the chemical indicators of organic contamination in all samples, probably because the values of microbial counts were not high enough to modify them. It should be noted that the same pattern has not been observed for the quantitative and qualitative microbiological parameters. Indeed, should be of concern the finding that a large number of non-carbonated and carbonated water sampled from coolers revealed a bacteria count higher than the limits stated for TVC. Moreover, contamination with *Escherichia coli *and *Enterococcus *spp. were not observed in any of the tap and dispensers water samples. The absence of these microorganisms, considered to represent an indicator of faecal contamination, renders the water satisfactory and safe with no health implications. The finding observed in the present study regarding the presence of a faecal indicator bacterium was in accordance with similar studies recently conducted [10,12]. Indeed, in water from coolers *Escherichia coli *and *Enterococcus *spp. were absent [10,12] and *Pseudomonas aeruginosa *has been detected in 24.1% of the water samples [10]. Furthermore, in contrast in a survey conducted in Canada on the microbiological quality of water from coolers located in residences and workplaces with respectively 28% and 36% of the collected samples contaminated by at least one coliform or indicator bacterium and/or one pathogenic bacterium [9].

In addition, we were interested to determine whether the tap water used was responsible for the contamination of the water dispensed by coolers. None of the tap water samples had a bacterial count higher than the water coolers and none of the samples were contaminated with coliforms. Thus, tap water was not directly responsible of water coolers contamination. These findings suggest that the contamination may be caused by the accumulation of small quantity of microorganisms from tap water or from faucet surface which are concentrated at filters.

It was interesting to find out that the results of the statistical analysis indicated that strongly and highly significant differences in quality and quantity of the microbiological parameters between the water coolers samples and the tap water samples. Indeed, the aerobic plate counts were higher in the coolers compared with the tap water and *Pseudomonas aeruginosa *was more frequently detected in the non-carbonated and carbonated water coolers samples than in those of tap water. These findings are in accordance with the two already mentioned studies, since the aerobic plate counts was higher in coolers compared with spring water [10] and a significantly higher proportion of water cooler samples resulted contaminated than tap water [9]. Therefore, a periodic adequate disinfection of water dispensers had to be indicated in order to keep the level of microbiological contamination under control. The validity of this recommendation is supported by the results of a study that showed that the periodic application of hydrogen peroxide (3%) of microfiltered water dispensers led to a reduction in the concentrations of *Pseudomonas aeruginosa *and to obtain water with bacteria counts conforming to Italian regulations for drinking water [12]. Furthermore, the data from this study demonstrated that no significant differences in bacterial counts occur between the non-carbonated and carbonated water in relation with the time since the last filter was substituted.

## Conclusion

The data presented here raise concern about the microbiological quality of the drinking water plumbed in water coolers and highlights the importance of adopting appropriate monitoring system with changing filters according to their use and the disinfection of the water in order to prevent or to diminish the chances of contamination of this water source.

## Competing interests

The authors declare that they have no competing interests.

## Authors' contributions

GL designed the study, was responsible for the data collection, and contributed to the interpretation of the data; IC, AA, CA, and DA collected the data and performed the laboratory analysis; IFA performed the statistical analysis, the interpretation of the data, and wrote the article. All authors have read and approved the final version of the manuscript.

## Supplementary Material

Additional file 1 **Table S1**. Microbiological characteristics of the samples of drinking water dispensed by the sampled water from coolers and tap according to the Italian legislation. Click here for file
