# Supplementary material for: Microbiological quality of drinking water from dispensers in Italy
Source: BMC Microbiol. 2010 Jan 26;10:19. doi: 10.1186/1471-2180-10-19 (PMC2824693; doi:10.1186/1471-2180-10-19)
Supplement: Additional file 1 — Table S1. Microbiological characteristics of the samples of drinking water dispensed by the sampled water from coolers and tap according to the Italian legislation. [file 1471-2180-10-19-S1.PDF]

Table 1. Microbiological characteristics of the samples of drinking water dispensed by the sampled water from coolers and tap according to the Italian legislation

**Source of water**	**No. of samples**	**TVC 22°C (CFU/mL)**		**TVC 37°C (CFU/mL)**		***Enterococcus* spp. (CFU/250 mL)**	***Escherichia coli* (CFU/250 mL)**	***Pseudomonas aeruginosa* (CFU/250 mL)**		**Other microorganisms (CFU/250 mL)**	
		**No. (%) of positive samples**	**Mean (range) concentration**	**No. (%) of positive samples**	**Mean (range) concentration**	**No. (%) of positive samples**	**No. (%) of positive samples**	**No. (%) of positive samples**	**Mean (range) concentration**	**No. (%) of positive samples**	**Mean (range) concentration**
**Tap water**	30	7 (18.4)	102.9 (0-1668)	7 (18.4)	86.3 (0-1664)	0	0	1 (3.3)	2	*Pseudomonas* spp. 6 (20)<br>*Pasteurella* spp. 1 (3.3)	48.3 (9-188)<br>15
**Non-carbonated**	38	30 (78.9)	569.7 (0-428)	34 (89.5)	331.8 (0-3016)	0	0	11 (28.9)	1.7 (0-25)	*Pseudomonas* spp. 19 (50)<br>*Stenotrophomonas* spp. 5 (13.2)<br>*Pasteurella* spp. 4 (10.5)<br>*Enterobacteria* spp. 3 (7.9)<br>*Flavobacterium* spp. 1 (2.6)	241.5 (2-1800)<br>606.4 (10-1620)<br>115.8 (10-400)<br>6 (1-15)<br>1400
**Carbonated**	38	36 (94.8)	542.1 (0-4140)	37 (97.4)	355.9 (0-1874)	0	0	9 (23.7)	2.5 (5-32)	*Pseudomonas* spp. 19 (50)<br>*Pasteurella* spp. 5 (13.2)<br>*Enterobacteria* spp. 4 (10.5)<br>*Stenotrophomonas* spp. 4 (10.5)<br>*Flavobacterium* spp. 1 (2.6)	137.2 (9-188)<br>105.8 (5-350)<br>5.5 (5-7)<br>930.8 (2-3100)<br>35

TVC: Total Viable Count
CFU: Colony Forming Units
